# Long-Term Outcomes of Pediatric Enterovirus Infection in Taiwan: A Population-Based Cohort Study

**DOI:** 10.3389/fped.2020.00285

**Published:** 2020-06-12

**Authors:** Jui-Ju Tseng, Chien-Heng Lin, Ming-Chih Lin

**Affiliations:** ^1^Children's Medical Center, Taichung Veterans General Hospital, Taichung, Taiwan; ^2^Institute of Clinical Medicine, National Yang-Ming University, Taipei, Taiwan; ^3^Department of Medical Research, Taichung Veterans General Hospital, Taichung, Taiwan; ^4^School of Medicine, National Yang-Ming University, Taipei, Taiwan; ^5^Department of Food and Nutrition, Providence University, Taichung, Taiwan

**Keywords:** enterovirus, ADHD, epilepsy, allergic disease, NHIRD

## Abstract

**Introduction:** The major burden of diseases in childhood has shifted from infectious diseases to chronic health conditions in recent decades. Although the rates of infectious diseases have decreased, the incidence of chronic diseases stemming from infectious agents continues to grow. Enterovirus is a major infectious disease of childhood and has been linked to numerous chronic diseases. We analyzed population-based data from Taiwan's National Health Insurance Research Database (NHIRD) to investigate the correlations between enterovirus infection and major chronic health conditions in children.

**Method:** Children diagnosed with enterovirus (EV) infection during 1999–2003 were identified from the Longitudinal Health Insurance Database 2000 (LHID 2000), a subdataset of Taiwan's National Health Insurance Research Database (NHIRD). A total of 14,168 patients were selected after excluding patients with existing chronic diseases and missing data. Another 14,168 children matched by age and sex were selected as the control group. Five primary outcomes, including attention deficit and hyperactivity disorder (ADHD), epilepsy, asthma, allergic rhinitis, and atopic dermatitis, were recorded.

**Results:** The risks of ADHD, asthma, allergic rhinitis, and epilepsy were significantly increased in the EV group compared with the control group. The risk of atopic dermatitis was significantly increased in the crude model. However, there were no significant differences in the adjusted model. The risks of ADHD, asthma, allergic rhinitis, and epilepsy were also significantly increased in patients with severe EV infection compared with patients with non-severe EV infection.

**Conclusion:** Chronic diseases, such as ADHD, epilepsy, asthma, allergic rhinitis, and atopic dermatitis were shown to be associated with enterovirus infection during childhood. EV infection during early childhood might have long-term public health implications and thus prevention strategies should be implemented.

## Introduction

The major burden of disease in children and adolescents has shifted from infectious diseases to chronic health conditions in recent decades (1, 2). Although rates of infectious diseases have decreased, it appears that chronic diseases stemming from infectious agents are on the rise. Infections may simply be the first misstep along a continuum from health to long-term disability and diseases (3).

Enteroviruses are among the most common infectious pathogens in infants and children. They are associated various clinical presentations from mild to severe illness, including non-specific febrile illness, mucosa-cutaneous presentation (herpangina, hand-foot-mouth disease, conjunctivitis, and various exanthems), CNS involvement (aseptic meningitis, encephalitis, poliomyelitis, Guillain-Barré syndrome, etc.), heart infection (myopericarditis), neonatal infection, and newly recognized clinical syndrome (asthma exacerbation secondary to EV D68 and Eczema Coxsackium) (4, 5). Enterovirus 71 (EV71) is responsible for a number of large epidemics of hand, foot, and mouth disease in children, and in rare cases can lead to a serious complication known as EV71 neurological disease (6). The largest and most severe outbreak of EV71 in Taiwan occurred in 1998 (7). Mortalities due to enterovirus infection decreased following the introduction of national stage-based management guidelines program in 2000 (8). However, long-term sequelae, particularly neurodevelopment, and cognitive function, remain a major concern in the management of this disease. Besides neurological sequelae, enterovirus infection has been shown to have other long-term effects resulting in common health problems, such as allergy diseases, autoimmune diseases, and diseases involving other systems, and for this reason enterovirus infection has become a major public health issue in children (5, 9).

This study investigated the relationship between enterovirus infection and common health problems in children from a population-based perspective (10).

## Method

### Data Source

The Longitudinal Health Insurance Database (LHID) is a subdataset of the National Health Insurance Research Database (NHIRD), which maintains anonymous claims data from Taiwan's National Health Insurance (NHI) program. The LHID 2000 contains all longitudinal claims data of 1,000,000 individuals randomly sampled from the registration data of beneficiaries of the NHI program during the period 1996–2000.

The ambulatory care expenditures by visit (CD) files and the inpatient expenditures by admission (DD) files were the main source of data used in the analysis. The LHID 2000 database used in this study provided medical information of those 1,000,000 NHI beneficiaries through the end of 2013.

### Study Population and Study Design

**Figure 1:**
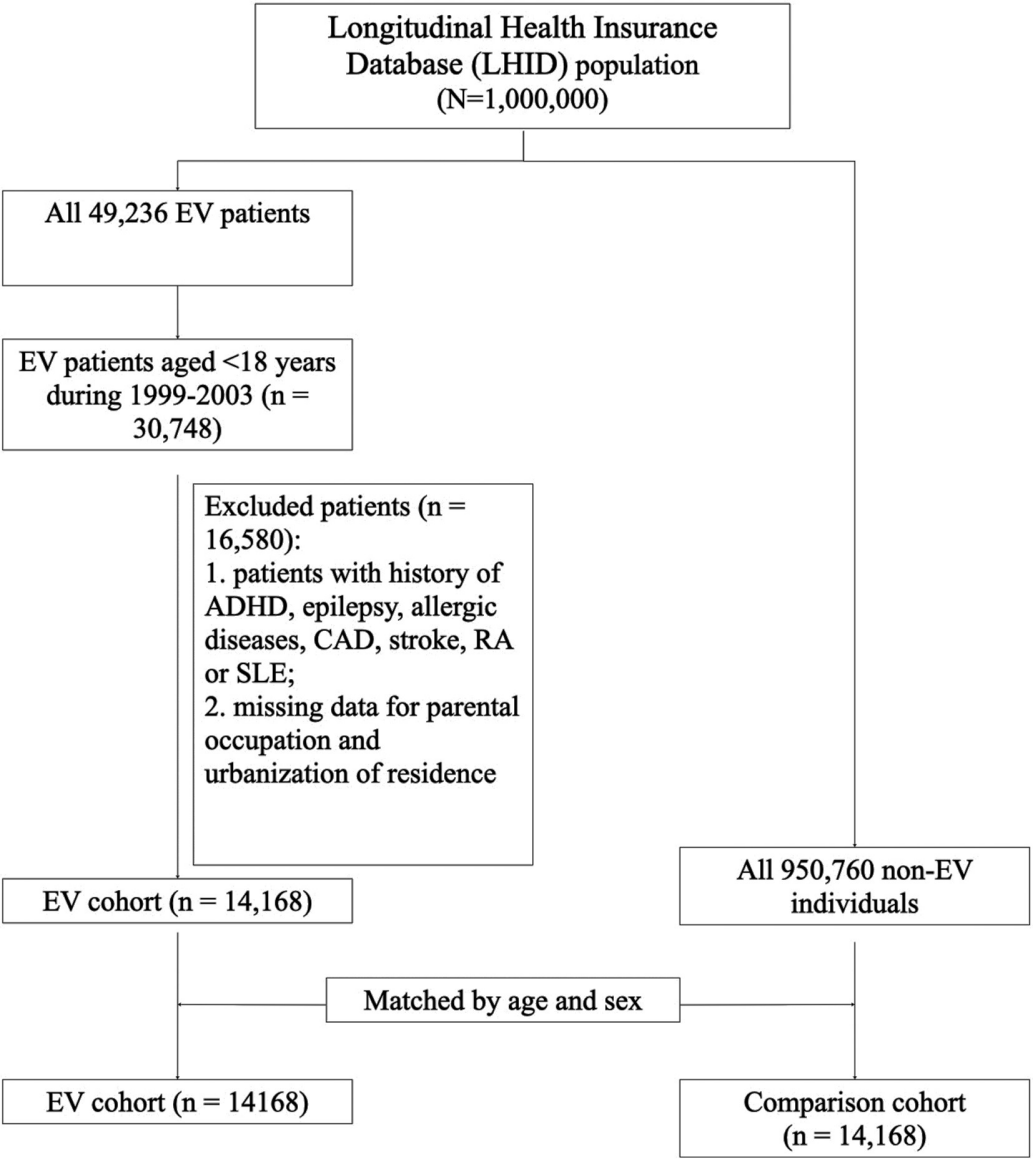
Flowchart of study design and study population selection.

The flow chart of the study design and selection of study subjects is shown in Figure 1. Since the cases of EV infection surged due to the endemic outbreak in 1998 in Taiwan, we chose to study since 1999 for clearer disease status definition. This is a claim data study. The basis of diagnosis is physicians' coding. International Classification of Diseases, 9th Revision, Clinical Modification (ICD-9-CM) was used by NHI of Taiwan during the study period. Symptomatic enterovirus infection (herpangina, hand-foot-mouth disease, enterovirus infection with CNS diseases) was defined as patients coded as ICD-9-CM 047, 048, 049, or 074. For the period 1999–2003, 49,236 patients with enterovirus (EV) infection (ICD-9-CM 047-049, 074) were identified from the LHID2000. Children aged <18 years were selected. Individuals diagnosed with attention deficit and hyperactive disorder (ADHD), epilepsy, atopic diseases, coronary artery disease (CAD), stroke, rheumatoid arthritis (RA), or systemic lupus erythematosus (SLE) before the diagnosis of enterovirus were excluded. Subjects with missing data related to parental occupation and urbanization were also excluded. Finally, a total of 14,168 subjects were included in the study. EV infection was further categorized according to level of severity based on the admission to hospital with diagnosis of EV infection. Subjects who were not hospitalized for EV infection were categorized as having a non-severe EV infection. Subjects who were hospitalized for EV infection were categorized as having a severe EV infection. The control group comprised individuals who were not diagnosed with EV infection during 1999–2003 matched by age and sex at a ratio of 1:1. Follow-up was terminated in each cohort when: the study subjects withdrew from the NHI program, a major outcome such as ADHD, epilepsy, asthma, allergic rhinitis, or atopic dermatitis occurred, or until December 31, 2013 was reached, as this was the end of the study period.

Five major outcomes were observed. The events were defined from CD and DD by ICD-9-CM codes. RA and SLE were defined from EV. Major outcomes were as follows: ADHD (ICD-9-CM 314), epilepsy (ICD-9-CM 345), asthma (ICD-9-CM 493), allergic rhinitis (ICD-9-CM 477), and atopic dermatitis (ICD-9-CM 691). Demographic variables including age, sex, parental occupation, and urbanization were collected as the control variables. Parental occupation was classified as “white-collar” for those working most hours indoors, “blue-collar” for those working most hours outdoors or involving industrial labor, and “others,” which included retirees and those with no occupation. Urbanization was categorized into four levels with level 1 defined as the highest degree of urbanization and level 4 defined as the lowest (11).

The study protocol was approved by the Institutional Review Board of Taichung Veterans General Hospital (IRB TCVGH No. CE17178A-2).

### Statistical Analysis

SAS 9.4 (SAS Institute Inc. Cary, NC, USA.) was used for data retrieval and data analysis. The descriptive statistical analysis of the EV and control groups, mean and standard deviation was performed to describe the continuous variables, such as age, number and, percentage were used to describe the categorical variables, such as gender, place of residence, and paternal occupation. The differences in the distribution of variables between the EV and control group were compared. *T*-test was used for comparing the continuous variables. Chi-square test was used for comparing categorical variables. The incidence of each disease was calculated using the incidence density. For assessing the risk of major outcomes after EV infection, the Kaplan-Meier method, and Log-Rank test were used for survival analysis. Cox regression models were used to control for confounding factors.

## Results

The study population consisted of 14,168 children diagnosed with EV infection and 14,168 children without EV infection. Table 1 shows the characteristics of the cohort and control group. The average age was 3.6 (*SD*: 2.9) years old in the EV group and 3.8 (*SD*: 3.0) years old in the control group. There was a significantly higher percentage of parents who were white-collar workers in the EV cohort group compared with the control group (cohort vs. control: 64.3 vs. 59.1%, *p* < 0.0001).

**Table 1:** Demographic characteristics of study population.

**Variable**	**With EV**	**Without EV**	***p*-value**
	***n* = 14,168**	***n* = 14,168**	
Age group (years)^*^			0.99
<6	11,551 (81.5)	11,551 (81.5)	
≥6	2,617 (18.5)	2,617 (18.5)	
Sex			0.99
Female	7,008 (49.5)	7,008 (49.5)	
Male	7,160 (50.5)	7,160 (50.5)	
Parental occupation			0.001
White-collar	9,107 (64.3)	8,377 (59.1)	
Blue-collar	3,135 (22.1)	3,431 (24.2)	
Others	1,926 (13.6)	2,360 (16.7)	
Urbanization of residence			0.303
1 (highest)	4,078 (28.8)	4,151 (29.3)	
2	4,222 (29.8)	4,304 (30.4)	
3	2,801 (19.8)	2,708 (19.1)	
4+ (lowest)	3,067 (21.6)	3,005 (21.2)	

* *t-test*. *EV, enterovirus*.

**Figure 2:**
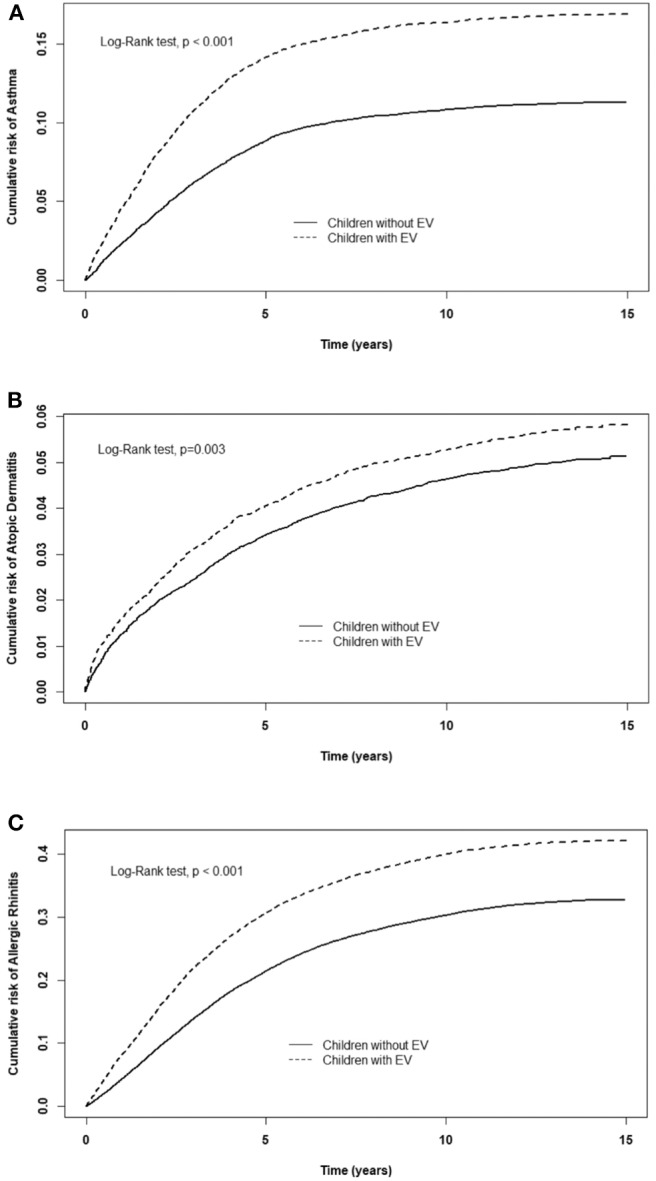
Cumulative risk of allergic diseases for children with or without EV infection. **(A)** asthma, **(B)** allergic rhinitis, **(C)** atopic dermatitis.

**Figure 3:**
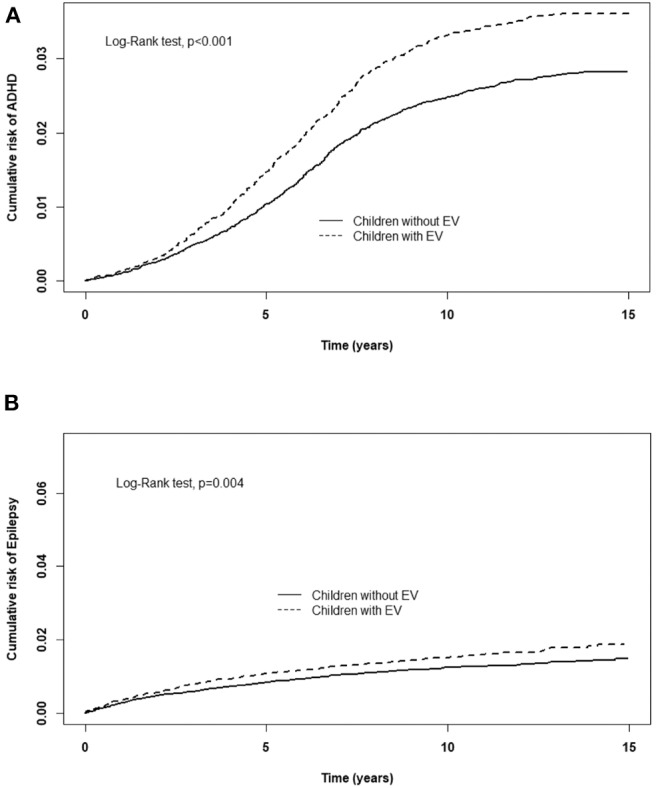
Cumulative risk of CNS sequelae for children with or without EV infection. **(A)** ADHD, **(B)** Epilepsy.

Table 2 displays the incidence rate and hazard ratio (HR) of 5 major events in the EV cohort group and control group. The incidence of ADHD was 28.0/10,000 person-years in the EV cohort group and 21.3/10,000 person-years in the control group. After adjusting for age, sex, paternal occupation, and urbanization level of residence, the EV cohort group had a 1.25 times greater risk for ADHD compared with the control group (HR = 1.25, 95%CI-1.11–1.41). The incidence rates of epilepsy were 13.7/10,000 person-years and 10.7/10,000 person-years in the EV cohort and control group, respectively. After adjusting for confounding factors, there was a 1.25 times higher risk for epilepsy in the EV cohort group compared with the control group. Three major allergic diseases were analyzed in this study: asthma, allergic rhinitis, and atopic dermatitis. The incidence of asthma was 150/10,000 person-years in the EV cohort group and 93.0/10,000 person-years in the control group. The risk of asthma was higher in the EV cohort group after adjusting for confounders (HR = 1.49, 95%CI = 1.41–1.58). The incidence of allergic rhinitis in the EV cohort group was 1.37 times higher than that of the control group (incidence rate = 462/10,000 person-years vs. 316/10,000 person-years, respectively). After adjusting for confounding factors, there was a 1.37 times higher risk for allergic rhinitis in the EV cohort group (HR = 1.37, 95%CI = 1.33–1.42). The incidence rates of atopic dermatitis were 45.6 and 39.3/10,000 person-years, respectively. However, after adjusting for confounders, there was no significant difference in the risk of atopic dermatitis between the two groups (HR = 1.09, 95%CI = 0.99–1.19). The cumulative risks for the five major events in the EV cohort group and control group were compared (Figures 2, 3).

**Table 2:** The incidence rate and hazard ratio (HR) of 5 major events in EV cohort and control group.

**Outcomes**	**With EV**			**Without EV**			**Crude HR**	Adjusted HR^*^
							**(95% CI)**	**(95% CI)**
	**Event**	**PYs**	**Rate**	**Event**	**PYs**	**Rate**		
ADHD	497	177,328	28.0	658	308,504	21.3	1.30 (1.16–1.46)	1.25 (1.11–1.41)
Epilepsy	245	178,880	13.7	331	310,557	10.7	1.27 (1.08–1.50)	1.25 (1.06–1.47)
Asthma	2,343	156,650	150	2,663	286,305	93.0	1.56 (1.48–1.65)	1.49 (1.41–1.58)
Allergic rhinitis	5,812	125,691	462	7,677	243,124	316	1.42 (1.37–1.47)	1.37 (1.33–1.42)
Atopic dermatitis	791	173,414	45.6	1,188	302,104	39.3	1.14 (1.05–1.25)	1.09 (0.99–1.19)

* *Model adjusted for age, sex, parental occupation, and urbanization of residence*. *ADHD, Attention Deficit/Hyperactivity Disorder; EV, enterovirus; HR, hazard ratio; PYs, person-years; Rate, incidence rate, per 10,000 person-years*.

**Figure 4:**
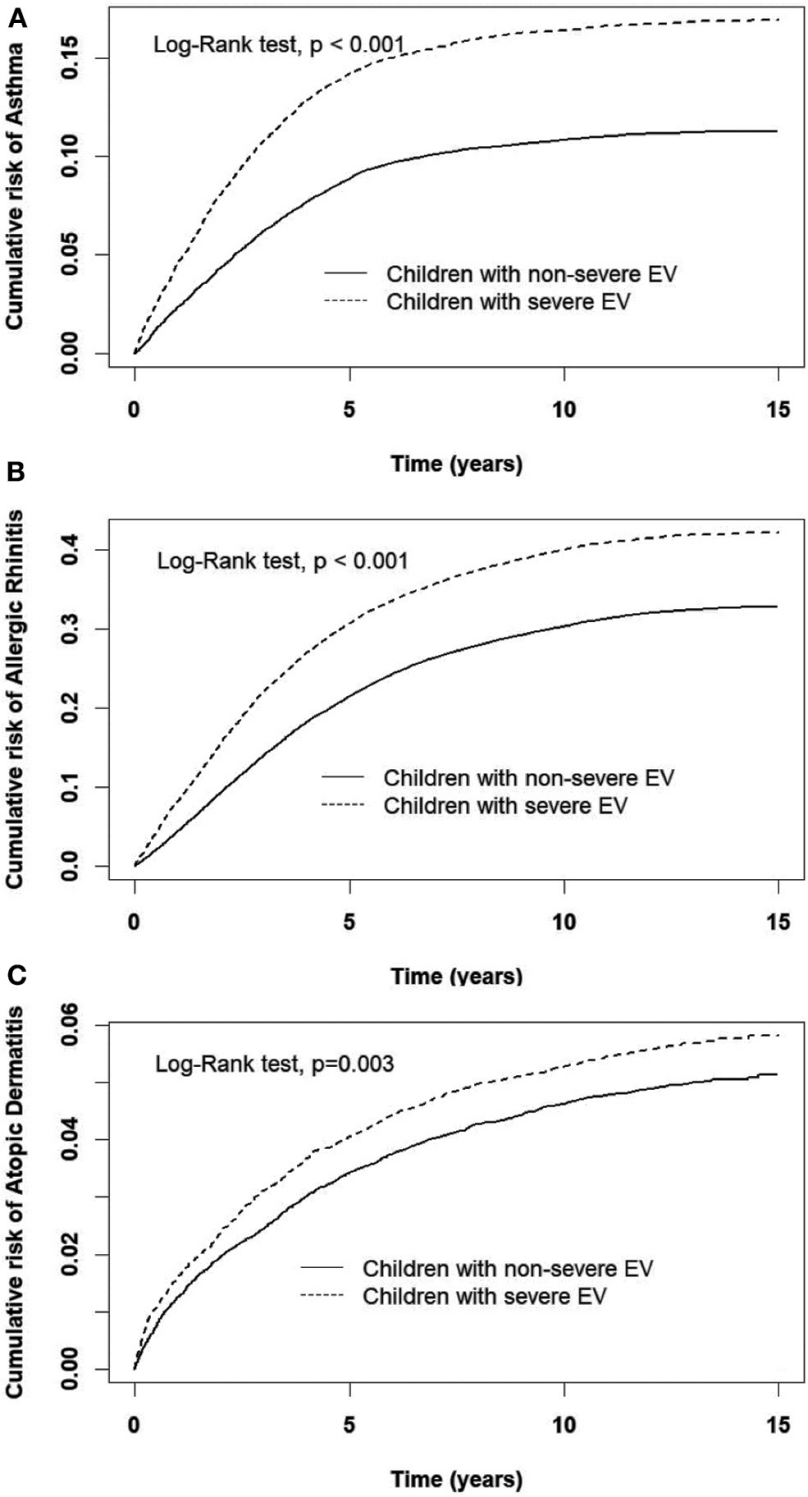
Cumulative risk of allergic diseases for children with severe and non-severe EV infection. **(A)** asthma, **(B)** allergic rhinitis, **(C)** atopic dermatitis.

**Figure 5:**
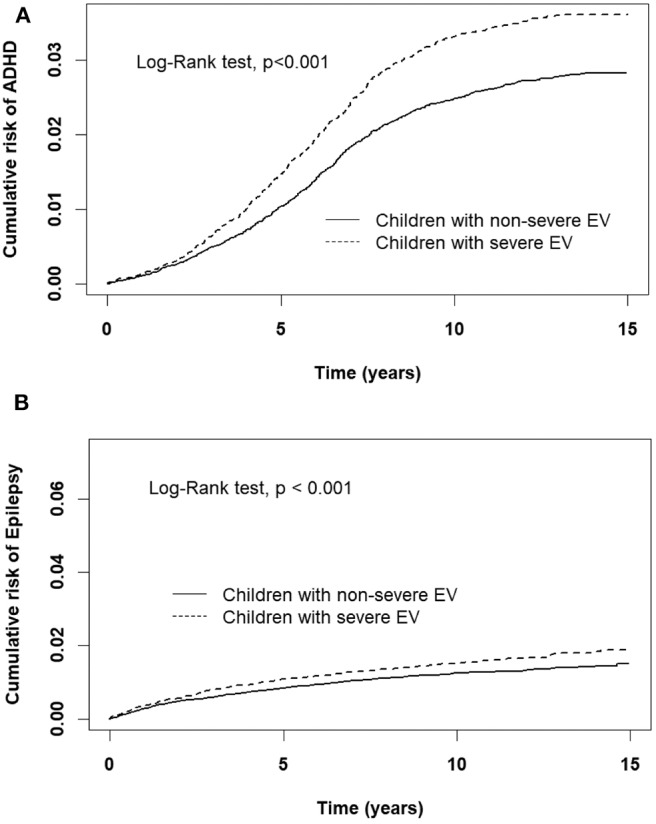
Cumulative risk of CNS sequelae for children with severe and non-severe EV infection. **(A)** ADHD, **(B)** Epilepsy.

A comparison of the risks of the five major events between patients with severe or non-severe EV infection was demonstrated in Table 3. With regard to neurologic diseases, the incidence and risk of ADHD between the severe EV and non-severe EV patients showed no difference (incidence rate = 34.2 vs. 27.4/10,000 person-years, respectively) (HR = 1.12, 95% CI = 0.84–1.47). The incidence of epilepsy was 29.5/10,000 person-years in patients with severe EV infection and 12.1/10,000 person-years in patients with non-severe EV infection. After adjusting for age, sex, paternal occupation, and urbanization of residence, compared with non-severe EV patients, severe EV patients had a 2.36 times greater risk for epilepsy (HR = 2.36, 95%CI = 1.62–3.24). Comparing the risk of allergic diseases between the two groups, there was no difference in risk of atopic dermatitis (HR = 1.10, 95%CI = 0.88–1.38), but there was a higher risk of asthma (HR = 1.33, 95%CI = 1.18–1.50) and allergic rhinitis (HR = 1.17, 95%CI = 1.08–1.28) in patients with severe EV infection. The cumulative risks for the five major events were compared between the severe EV and non-severe infection groups (Figures 4, 5).

**Table 3:** The risk of five major events for children with or without severe EV infection^*^.

**Outcomes**	**Severe EV71**			**Non-severe EV71**			**Crude HR**	Adjusted HR^*^
							**(95% CI)**	**(95% CI)**
	**Event**	**PYs**	**Rate**	**Event**	**PYs**	**Rate**		
ADHD	57	16,680	34.2	440	160,647	27.4	1.27 (0.96–1.67)	1.12 (0.84–1.47)
Epilepsy	49	16,587	29.5	196	162,293	12.1	2.45 (1.79–3.35)	2.36 (1.72–3.24)
Asthma	306	13,762	222	2,037	142,888	143	1.55 (1.37–1.75)	1.33 (1.18–1.50)
Allergic rhinitis	617	10,915	565	5,195	114,776	453	1.24 (1.14–1.35)	1.17 (1.08–1.28)
Atopic dermatitis	87	16,190	53.7	704	157,224	44.8	1.21 (0.97–1.52)	1.10 (0.88–1.38)

* *Model adjusted for age, sex, parental occupation, and urbanization of residence*. *ADHD, Attention Deficit/Hyperactivity Disorder; EV, enterovirus; HR, hazard ratio; PYs, person-years; Rate, incidence rate, per 10,000 person-years*. *Rate: per 10,000 person-years*.

## Discussion

The major burden of public health conditions associated with infants, children, and adolescents has changed dramatically over recent decades (1, 2, 10). The impacts of serious childhood infectious diseases have declined gradually; however, the incidence of chronic health conditions has risen steadily. The reasons for these changes are thought to be due to improved nutrition and sanitation, implementation of childhood immunization programs for preventing severe pathogens, medical advances in both treating primary diseases and managing serious complications, and better public awareness of chronic health conditions (1). Much of the rise in chronic health conditions can be attributed to four common diseases, namely asthma, obesity, mental health disorders, and neurodevelopmental disorders. There is a growing recognition that certain chronic diseases stem from infectious diseases. Recent advances in diagnostic techniques have enabled researchers to prove a causal relationship between various infectious agents and chronic diseases (3).

According to long-term data gathered by Taiwan's CDC, the number of outpatients and emergency visits due to enterovirus, i.e., herpangina or hand-foot-mouth disease, increases during late March, peaks around mid-June, and then declines gradually. Children under 5 years old are more prone to enterovirus infection with severe complications, resulting in case-fatality rates ranging from 1.3 to 33.3%. A previous study also reported more than 93% of cases with severe enterovirus infection were found in children who were younger than 4 years old, and epidemics of enterovirus occur every 2–3 years due to the accumulation of a new population of susceptible individuals (7). Enterovirus 71 (EV71) is a major etiological pathogen responsible for a number of severe diseases and complications (6). Besides hand-foot-mouth disease (HFMD) and herpangina, this virus can be potentially complicated with severe neurological complications resulting in mortality of young children. In Taiwan, the largest and most severe enterovirus 71 epidemic to date occurred in 1998, and almost all of the patients with cardiopulmonary failure died (7, 12, 13). In 2000, a national stage-based management program was developed to improve survival and in subsequent years the case-fatality rate has greatly improved (8, 14). However, despite high level PICU support, enterovirus can still cause significant mortality, morbidity, and long-term sequelae (15). Due to the increased survival rate following the introduction of the national stage-based management guidelines program (8), more patients infected with enterovirus survived; however, the long-term sequelae, particularly neurodevelopment and cognitive function, remain a major concern in the management of this disease (8, 16). In addition to neurological sequelae, enterovirus infection is thought to have a long-term impact on various common health problems, such as allergic diseases, and diseases involving other systems, and thus enterovirus infection is becoming a major public health issue in children (5, 9).

In this study, we compared children with and without EV infection, and found a higher risk of CNS and allergic sequelae in the former group. There was a higher risk of ADHD, epilepsy, asthma, and allergic rhinitis in children with severe EV infection compared with children with non-severe EV infection. The cumulative risks for ADHD, epilepsy, asthma, allergic rhinitis, and atopic dermatitis were significantly increased in children with EV infection, especially in children with severe EV infection in the follow-up period.

Viral CNS infections during childhood have been documented to be associated with later development of long-term neurologic sequelae, such as limb weakness, cranial nerve palsy, cerebral palsy, non-affective psychotic illness, cognitive impairment, attention deficit hyperactivity disorder (ADHD), learning disability, and epilepsy (16–20). Enterovirus has been reported as one of the major causes of viral CNS infection (21, 22). Several studies have assessed the long-term neurological sequelae secondary to enterovirus CNS involvements (16, 23–25). Even when CNS manifestations are totally resolved, psychiatric problems may be noted when children enter school (23). In our study, the risks of ADHD and epilepsy were increased, especially in children with severe EV infection. This implies that early recognition of psychiatric and cognitive problems and early intervention are crucial, especially for children who are infected with enterovirus at a young age. Patients with severe EV infection are of particular concern in this regard. In the present study, severe EV infection identified by hospital admission for EV infection is a major public health issue that needs to be urgently addressed.

Allergic diseases are increasing in incidence in the general population and their symptoms can last for a long time causing negative effects on quality of life (26, 27). Moreover, allergic diseases are a major social burden due to school absence and results in significant medical expenditure worldwide (28–31). Multiple environmental factors also increase the risk of allergic diseases, including increased use of antibiotics, improved hygiene, smaller family sizes, use of volatile products (32, 33), and personal factors (34) (obesity and resident lung microbiome). The innate immune response to viral infections was reported to be an important factor for inducing allergic disease during childhood. Rhinovirus and respiratory syncytial virus-induced respiratory infection were shown to be significantly correlated with an increased occurrence of asthma (35–38). Human enterovirus has been reported to be correlated with development of allergic disease (39–41). Enterovirus D68 (EV-D68) was identified in 1962, but was previously rare until an outbreak occurred in the United States in 2014. EV-D68 can cause severe respiratory disease similar to human rhinovirus, and exacerbates the severity of symptoms in children with asthma and wheezing (5, 42). Furthermore, previous studies reported an increased seroprevalence of enterovirus 71 IgE antibodies and lowered anti-echovirus antibodies in asthmatic children with HFMD (43, 44). An elevated level of circulating follicular helper T cells in children with HFMD has been also reported (45). It is reasonable to postulate that enteroviruses influence subsequent risks of allergic diseases in children via the immune system. Recently, Lee et al. observed an increased risk of subsequently developing allergic dermatitis and allergic rhinitis in children who had previously been infected with herpangina and a decreased risk of subsequent occurrence of asthma in children with a prior diagnosis of HFMD compared to the general population, based on an analysis of a large population-based database (40). However, in our study, increased risks of asthma and allergic rhinitis were noted in children who had previously had an enterovirus infection compared to the general population. We also found an increased risk of asthma and allergic rhinitis in children who had been hospitalized with severe enterovirus infection compared to children with non-severe enterovirus infection. Nonetheless, there was only a borderline significant difference in risk of atopic dermatitis between the EV cohort and control group. A significantly increased cumulative risk of atopic dermatitis in the follow-up period was observed. The difference between our result and the finding of the aforementioned study might be due to the definitions of disease that were used.

Enteroviruses have been investigated for several decades. A previous study explored associations between the genotypes of enterovirus and the clinical spectrum, but the results showed considerable diversity between causative genotypes in each clinical presentation (15). Several chronic diseases such as type I DM (46), leukemia (47), tic disorders (48), autoimmune disease (49, 50), and cardiovascular diseases might be associated with enterovirus infections (9). While some of these disorders and diseases have been reported to have a strong causal relationship with enterovirus, such as type I DM, others have shown conflicting findings in the literature. Thus, taken together, the current body of available evidence indicates that enterovirus is closely linked with numerous chronic health conditions, and that for children, this pathogen may confer a considerable long-term health burden extending well into adulthood.

The main strength of this study is that it is the first epidemiological study demonstrating associations of common pediatric diseases with enterovirus infection using data from a nationally representative cohort with a long period of follow-up. Although the data used in this study were obtained from Taiwan's National Health Insurance Research Database (NHIRD), which is a large, population-based databased with highly reliable in diagnoses due to strict peer review process by qualified specialists, there is certain limitations. It does not contain data related to laboratory tests including viral isolation from tissue, viral serum titer or reverse transcription polymerase chain reaction (RT-PCR). Moreover, the causal relationship cannot be further explored in this study.

In conclusion, chronic diseases, such as ADHD, epilepsy, and allergic diseases were found to be associated with enterovirus infection during childhood. EV infection during early childhood might have long-term public health implications and thus prevention programs should be developed.

## Data Availability Statement

The datasets presented in this article are not readily available because data release is not allowed by the National Health Insurance Research Database. Requests to access the datasets should be directed to Dr. Chien-Heng Lin/epid@ms39.hinet.net.

## Ethics Statement

The studies involving human participants were reviewed and approved by Institutional Review Board of Taichung Veterans General Hospital. Written informed consent from the participants' legal guardian/next of kin was not required to participate in this study in accordance with the national legislation and the institutional requirements.

## Author Contributions

M-CL and C-HL designed the model and computational framework. C-HL analyzed the data and performed the calculations. J-JT wrote the manuscript with input from all authors. M-CL conceived the study and were in charge of overall direction and planning.

## Conflict of Interest

The authors declare that the research was conducted in the absence of any commercial or financial relationships that could be construed as a potential conflict of interest.
